# A nomogram based on 9-lncRNAs signature for improving prognostic prediction of clear cell renal cell carcinoma

**DOI:** 10.1186/s12935-019-0928-5

**Published:** 2019-08-05

**Authors:** Wen Jiang, Qing Guo, Chenghe Wang, Yu Zhu

**Affiliations:** 1 Department of Urology, Ruijin Hospital, School of Medicine, Shanghai Jiao Tong University, Shanghai, 200025 China; 2 Department of Pediatrics, Union Hospital, Tongji Medical College, Huazhong University of Science and Technology, 1277 Jiefang Avenue, Wuhan, 430022 China

**Keywords:** Survival, Prognosis, Nomogram, Long noncoding RNA (lncRNA), Clear cell renal cell carcinoma (ccRCC)

## Abstract

**Background:**

Abnormal expressions of long noncoding RNAs (lncRNAs) are very common in clear cell renal cell carcinoma (ccRCC), and some of these have been reported to be highly correlated with prognosis of ccRCC patients.

**Methods:**

“edgeR” AND “DEseq” R packages were used to explore differentially expressed genes (DEGs) between normal and tumor tissues of ccRCC samples from The Cancer Genome Atlas (TCGA). Univariable Cox survival analysis, robust likelihood-based survival model and multivariable Cox regression analysis were used to identify prognostic lncRNAs and construct lncRNAs signature. Finally, a graphic nomogram based on the lncRNAs signature was developed to predict 1-, 3- and 5-year survival probability of ccRCC patients by using rms R package.

**Results:**

8413 DEGs including 2740 lncRNAs and 4530 mRNAs were identified between normal and tumor tissues. 395 lncRNAs were found to be associated with prognosis of ccRCC patients (P < 0.05). Among these 395 prognostic lncRNAs, 9 key prognostic lncRNAs (RP13-463N16.6, CTD-2201E18.5, RP11-430G17.3, AC005785.2, RP11-2E11.9, TFAP2A-AS1, RP11-133F8.2, RP11-297L17.2 and RP11-348J24.2) were identified by using robust likelihood-based survival model. A 9-lncRNAs signature was constructed by using estimated regression coefficients of the 9 key prognostic lncRNAs. Results of χ^2^-test or Fisher’s exact test indicated that the 9-lncRNAs signature was significantly associated with clinicopathological characteristics such as tumor grade, T stage, N stage, M stage, TNM stage and survival outcome of ccRCC patients. Multivariate analysis showed that the 9-lncRNAs signature, age and M stage were independent prognostic factors. Finally, a graphic nomogram based on the lncRNAs signature, age and M stage was developed to predict 1-, 3- and 5-year survival probability of ccRCC patients by using rms R package.

**Conclusions:**

A 9-lncRNAs signature associated with prognosis of ccRCC patients was constructed and a promising prognostic nomogram based on the 9-lncRNAs signature was developed for 1-, 3- and 5-year OS prediction of ccRCC patients in this study.

## Background

Approximately 350,000 new cases of renal cell carcinoma (RCC) were diagnosed worldwide per year, and this cancer caused more than 140,000 deaths per year [1]. Clear cell renal cell carcinoma (ccRCC), which makes up 70% of kidney cancers, is the most common subtype of RCC [2]. The prognosis varies widely among ccRCC patients, 5-year survival rate of patients with localized disease was more than 90%, however 5-year survival rate of patients with distant metastasis was only 12% [3]. TNM [Tumor (T), Node (N), Metastasis (M)] staging system, developed and maintained by the International Union Against Cancer (UICC) and American Joint Committee on Cancer (AJCC), is the most commonly prognostic predictive system for ccRCC patients. However, TNM staging system always lacked accuracy for prognostic prediction of ccRCC patients as external tumor factors such as age, type of disease presentation, performance status, nuclear grading, and microscopic tumor necrosis were ignored [4].

Recently, several novel systems have been developed for prognostic prediction of ccRCC patients. For example, Stage, Size, Grade, and Necrosis (SSIGN) Score system, which was firstly reported in 2002 for outcome prediction of ccRCC patients, has been proved to have better predictive ability than TNM staging system [5]. Another staging system consisted of tumor grade, N stage and patients’ performance status could accurately distinguish RCC patients with different survival possibility [6]. However, these systems included parameters depended on subjective judgement of professional pathologist, and they made prediction easily even influenced by inter-observer variabilities. Therefore, more concise and practical tools are urged for improving prognostic prediction of ccRCC patients.

Recent years, emerging evidences have found that long noncoding RNAs (lncRNAs) play important roles in regulation of mRNA transcription and protein translation [7, 8]. Abnormal expressions of lncRNAs are frequent biological phenomena in tumor and closely associated with prognosis of tumor patients. For example, overexpression of lncRNA urothelial carcinoma associated 1 (UCA1), which could promote aggressiveness of cancer cell, is associated with prognosis of multiple tumor such as bladder cancer, colorectal cancer, gastric cancer and so on [9–12]. LncRNA metastasis-associated lung adenocarcinoma transcript 1 (MALAT1) was first discovered in lung cancer, and up-regulation of MALAT1 is negatively associated with survival time of lung cancer patients [13]. Up-regulation of MALAT1 was also found in RCC patients and could promote proliferation and invasion of RCC [14]. Another lncRNA titled metastatic renal cell carcinoma-associated transcript 1 (MRCCAT1) participated in activating p38-MAPK signaling by inhibiting NPR3 and could promote metastasis of ccRCC, and its up-regulation was associated with poorer outcome of ccRCC patients [15]. In summary, lncRNAs are important participants of various biological process and can be important biomarkers source of prognostic prediction and tumor targeted therapy.

In this study, a 9-lncRNAs signature associated with prognosis of ccRCC was identified by mining RNA-Seq data from The Cancer Genome Atlas (TCGA, https://tcga-data.nci.nih.gov/tcga/). Then, a nomogram based on this 9-lncRNAs signature was developed for improving prognostic prediction of ccRCC patients, and it will be a useful diagnostic tool for ccRCC patients in the future.

## Methods

### Data source and reprocessing

RNA-Seq data of ccRCC patients together with the corresponding clinicopathological data was obtained from TCGA. Ensembl IDs were annotated in the form of gene symbols and biotypes based on GENCODE project gene annotation file (version 22, GRCh38). Then, reads per kilobase per million mapped reads (RPKM) were transformed into transcripts per kilobase of exon model per million mapped reads (TPM) for data standardization. Because of huge numerical span of TPM values, the gene expressions of each gene were then presented in the form of log2(TPM + 1) and integrated into one matrix table.

### Identification of differentially expressed genes (DEGs)

In order to compare expression differences of genes between normal and tumor tissue, P value and fold change (FC) of each gene was generated by using “edgeR” and “DEseq” R packages, and genes with P value < 0.05 and | log_2_ FC| > 1 were defined as DEGs [16, 17]. However, expression levels of some DEGs were very low, these DEGs were further filtered out referring to criterion of previous research [18]. Finally, abundantly differentially expressed LncRNAs and mRNAs were separated from the remaining DEGs, and all patients were randomly divided into training group and testing group for the following analysis.

### Selection of prognostic lncRNAs

Then, univariable Cox survival analysis was employed to explore relationships between overall survival (OS) and the differentially expressed LncRNAs in training group. Parameters including hazard rate (HR) and P value were generated by using survival package in R environment. The lncRNAs with P value < 0.05 were selected as prognostic lncRNAs for the following screening.

### Identification of key prognostic lncRNAs

However, it was no suitable for establishment of risk score formula because of large number of prognostic lncRNAs. Then, a Robust likelihood-based survival model was used to identify the key prognostic lncRNAs of ccRCC patients [19]. The detail procedure was as followings:

1. All samples were randomly divided into two sets: the training set (2/3*N samples) and the validation set (1/3*N samples). Fit a lncRNA to the training set of samples and obtain the parameter estimate for the lncRNA. Then we evaluate log likelihoods with the parameter estimate and the validation set of samples. The evaluation was repeated for every lncRNA.
2. After 10 repetitions of the above procedure, we obtained 10 log likelihoods for each lncRNAs. The best lncRNA with the largest mean log likelihood was selected. Subsequently, the next best lncRNA were searched by evaluating every two-lncRNA model and an optimal one was selected with the largest mean log likelihood.
3. This forward lncRNA selection procedure was continued until fitting is impossible, and a series of models were got. Akaike’s information criterions (AICs) for all the candidate models were computed and an optimal model with the smallest AIC was selected.

After repeating procedure for 1000 times, key prognostic lncRNAs were finally selected out from the prognostic lncRNAs.

### Risk score formula establishment

Multivariable Cox regression analysis was used to generate estimated regression coefficients of key prognostic lncRNAs in the training group, and then a lncRNAs signature consisted of these lncRNAs was constructed by using respective coefficient. According to the risk score formula, risk score of each patient was calculated and the optimal risk score was determined by using survminer R package to cut patients of training group into low-risk group and high-risk group. Then, OS differences between low-risk group and high-risk group were compared by using Kaplan–Meier method. In order to test the sensitivity and specificity of the risk score formula, time-dependent dynamic receiver operating characteristic (ROC) curve area under the curve (AUC) values (1–10 years) were generated by using survival ROC R package [20]. Similarly, the formula was further applied in the testing group and training + testing group to validate its stability.

### Clustering analysis of key prognostic lncRNAs

According to the HRs of key prognostic lncRNAs, lncRNA associated with better prognosis (HR < 1) was defined as protective lncRNA and lncRNA associated with worse prognosis (HR > 1) was defined as risk lncRNA. A patient would get one risk factor if the expression of risk lncRNA in this patient > median expression of risk lncRNA or expression of the protective lncRNA in this patient < median expression of protective lncRNA. Then the number of risk factors was counted for every patient in training + testing set. Survival analysis was performed by using different number of risk factors as cut-off values.

### Prediction ability of the lncRNAs signature for localized ccRCC and advanced ccRCC

As we known, prognosis of ccRCC patients is different between localized ccRCC (stage I and II) and advanced ccRCC (stage III and IV). So, survival analysis was performed between localized ccRCC patients and advanced ccRCC patients by using Kaplan–Meier method, respectively. Meanwhile, ROC AUC value of 1–10-years survival was calculated to testing sensitivity and specificity of the lncRNAs signature.

### Drug response prediction

Because not all advanced ccRCC patients were sensitive to radiotherapy and chemotherapy, we used the lncRNAs signature to perform drug sensitivity prediction by R package “pRRophetic”. Common antitumor drugs of ccRCC such as axitinib, cisplatin, gemcitabine, pazopanib, sorafenib and temsirolimus were selected from the pharmacogenomics database “The Genomics of Drug Sensitivity in Cancer” (GDSC) (https://www.cancerrxgene.org/) [21, 22]. Half-maximal inhibitory concentration (IC50) of TCGA samples were estimated by ridge regression against the GDSC training set [23]. Tenfold cross-validation was used to evaluated prediction accuracy of estimated IC50. Mann–Whitney–Wilcoxon Test was used to test whether IC50 distributions of high risk group and low risk group were identical.

### Weighted gene co-expression analysis (WGCNA) and gene enrichment analysis

In order to explore relationships between lncRNAs signature and biologic functions of ccRCC, weighted gene co-expression network analysis (WGCNA) was employed to construct the gene co-expression modules among differentially expressed mRNAs [24]. The modules with the maximal absolute module significance associated with lncRNAs signature were selected out. Then, gene enrichment analysis of genes in the most lncRNAs signature related module was performed by using cluster Profiler R package [25].

### Relationship between risk score and clinicopathological characteristics

χ^2^-Test or Fisher’s exact test was employed to explore relationships between lncRNAs signature and clinicopathological characteristics such as age, tumor grade, TNM stage and so on. Because clinicopathological characteristics such as TNM stage are highly associated with prognosis of ccRCC patients, univariable and multivariable Cox regression analysis were performed to test whether the lncRNAs signature was independent of clinicopathological characteristics.

### Nomogram construction based on lncRNAs signature

Finally, a nomogram consisted of independent prognostic factors was constructed by employing rms R package. Separating capacity of the nomogram was tested by Harrell’s concordance index (C-index), and the calibration curves of the nomogram were constructed to test consistency between 1-, 3- and 5-year survival probability prediction based on the nomogram and actual observation.

## Results

### Data acquisition and DGEs identification

**Fig. 1:**
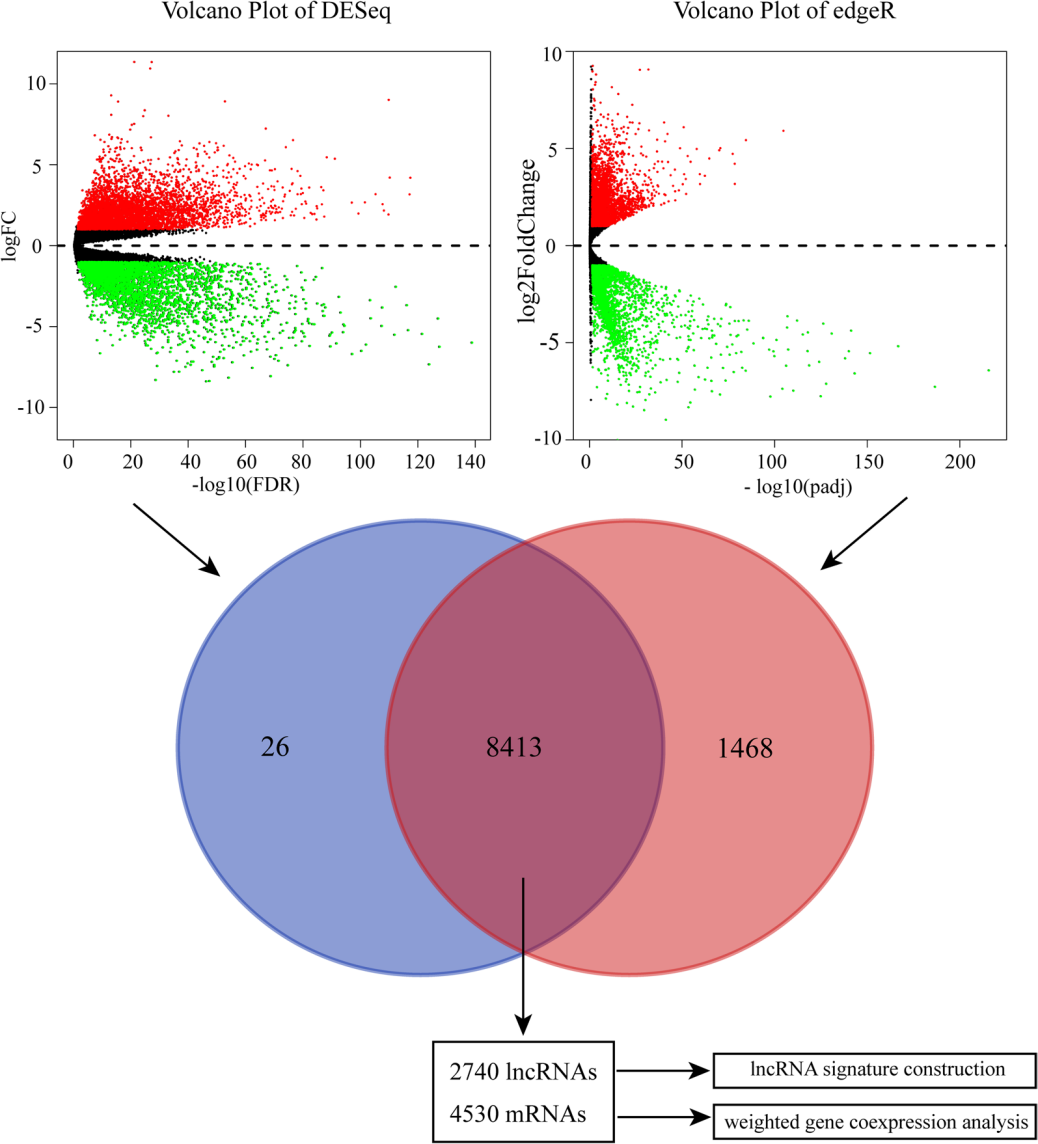
Identification of differentially expressed genes (DEGs) by using “edgeR” and “DEseq” R package

In total, 60,483 genes of 516 patient samples were re-annotation by using version 22 GENCODE project gene annotation file. After comparing expression level of genes between 72 normal tissue and 72 ccRCC, 8413 DGEs including 2740 lncRNAs and 4530 mRNAs were obtained (Fig. 1). After removing lncRNAs of low expression, 1062 abundantly expressed lncRNAs were finally selected out. Then, the 516 samples were randomly divided into a training group (258 samples) and a testing group (258 samples) for following analysis.

### Identification of key prognostic lncRNAs

**Fig. 2:**
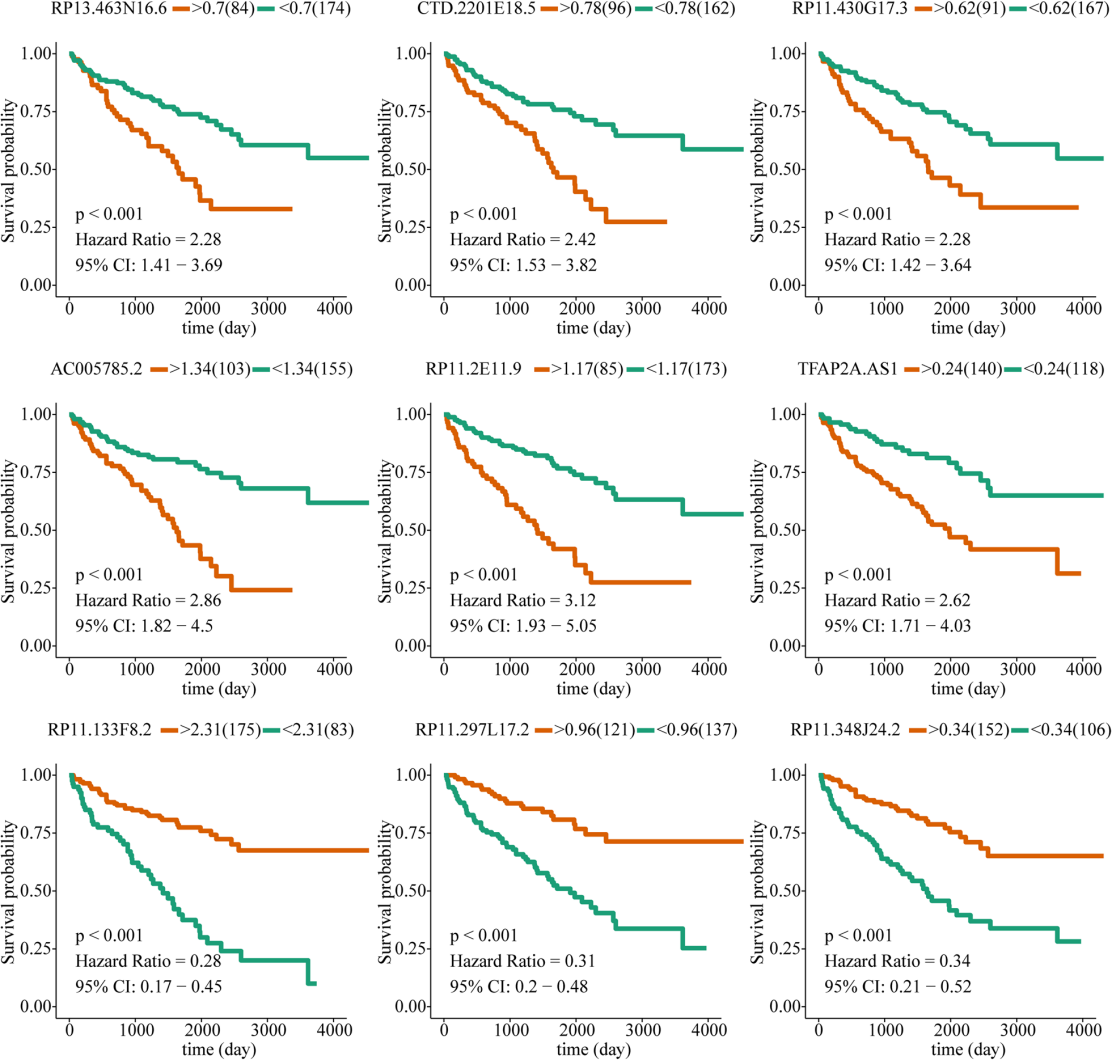
Survival analysis of 9 key prognostic lncRNAs by using optimal risk score as cut-off value

395 prognostic lncRNAs (P < 0.05) were identified by univariable Cox survival analysis in training group, and top 20 lncRNAs with least P value were presented in Table 1. Because number of prognostic lncRNAs is too much, 9 key prognostic lncRNAs were finally picked out from the 395 prognostic lncRNAs by using robust likelihood-based survival analysis (Table 2). Then, survival analysis was performed for the 9 lncRNAs, among these lncRNAs, 6 lncRNAs (RP13-463N16.6, CTD-2201E18.5, RP11-430G17.3, AC005785.2, RP11-2E11.9 and TFAP2A-AS1) were associated with worse prognosis, and the remaining 3 lncRNAs (RP11-133F8.2, RP11-297L17.2 and RP11-348J24.2) were associated with better prognosis (Fig. 2).

**Table 1:** The top 20 prognostic lncRNAs of ccRCC with least P value

lncRNA	HR	P value
RP11-133F8.2	0.71	9.2E−09
RP11-2E11.9	2.87	1.16E−08
APCDD1L-AS1	1.83	1.66E−08
TFAP2A-AS1	1.97	1.82E−08
RP4-555D20.2	2.02	3.05E−08
RP13-463N16.6	2.35	4.64E−08
LINC00941	1.80	1.26E−07
FIRRE	2.36	2.53E−07
CTD-2201E18.5	2.24	2.9E−07
CTD-2357A8.3	2.60	6.26E−07
RP11-430G17.3	2.80	7.88E−07
AC005785.2	2.29	8.77E−07
RP11-247A12.7	1.99	1.23E−06
RP11-297L17.2	0.40	2.3E−06
RP11-837J7.4	1.86	3.12E−06
CTD-2035E11.5	1.80	3.19E−06
RP11-462L8.1	1.60	3.62E−06
RP3-404F18.5	2.16	3.63E−06
RP11-384O8.1	1.82	4.04E−06
LINC00460	1.37	4.13E−06

**Table 2:** Key prognostic lncRNAs screened by using robust likelihood-based survival analysis

Gene	Log likelihood	AIC
RP13-463N16.6	407.3	816.61
CTD-2201E18.5	401.68	807.36
RP11-430G17.3	400.82	807.65
AC005785.2	398.93	805.87
RP11-2E11.9	393.36	796.72
TFAP2A-AS1	391.27	794.54
RP11-133F8.2	382.14	778.28
RP11-297L17.2	378.42	772.84
RP11-348J24.2	376.7	771.39

*AIC* Akaike’s information criterions

### Establishment of 9-lncRNAs risk score formula

Based on the estimated regression coefficients of the 9 lncRNAs, a risk score formula was finally developed. The risk score of each patient was calculated referring to the following formula:

$$\begin{aligned} {\text{Risk score}}\, & = \,\left( {0. 7 7 3 2 1\, \times \,{\text{relative expression of RP13}} - 4 6 3 {\text{N16}}. 6} \right)\, - \,\left( {0. 3 6 5 5 6\, \times \,{\text{relative expression of CTD}} - 2 20 1 {\text{E18}}. 5} \right)\, + \,\left( {0. 2 4 3 4 9\, \times \,{\text{relative expression of RP11}} - 4 30{\text{G17}}. 3} \right) \\ & + \,\left( {0. 3 7 8 3 9\, \times \,{\text{relative expression of AC}}00 5 7 8 5. 2} \right)\, + \,\left( {0. 7 4 4 2 5\, \times \,{\text{relative expression of RP11}} - 2 {\text{E11}}. 9} \right) + \,\left( {0.0 3 50 9\, \times \,{\text{relative expression of TFAP2A}} - {\text{AS1}}} \right) \\ & - \,\left( {0.0 1 5 2 4\, \times \,{\text{relative expression of RP11}} - 1 3 3 {\text{F8}}. 2} \right)\, - \,\left( {0. 5 1 7 7\, \times \,{\text{relative expression of RP11}} - 2 9 7 {\text{L17}}. 2} \right)\, - \,\left( {0. 8 5 4 3\, \times \,{\text{relative expression of RP11}} - {\text{ 348J24}}. 2} \right). \\ \end{aligned}$$

**Fig. 3:**
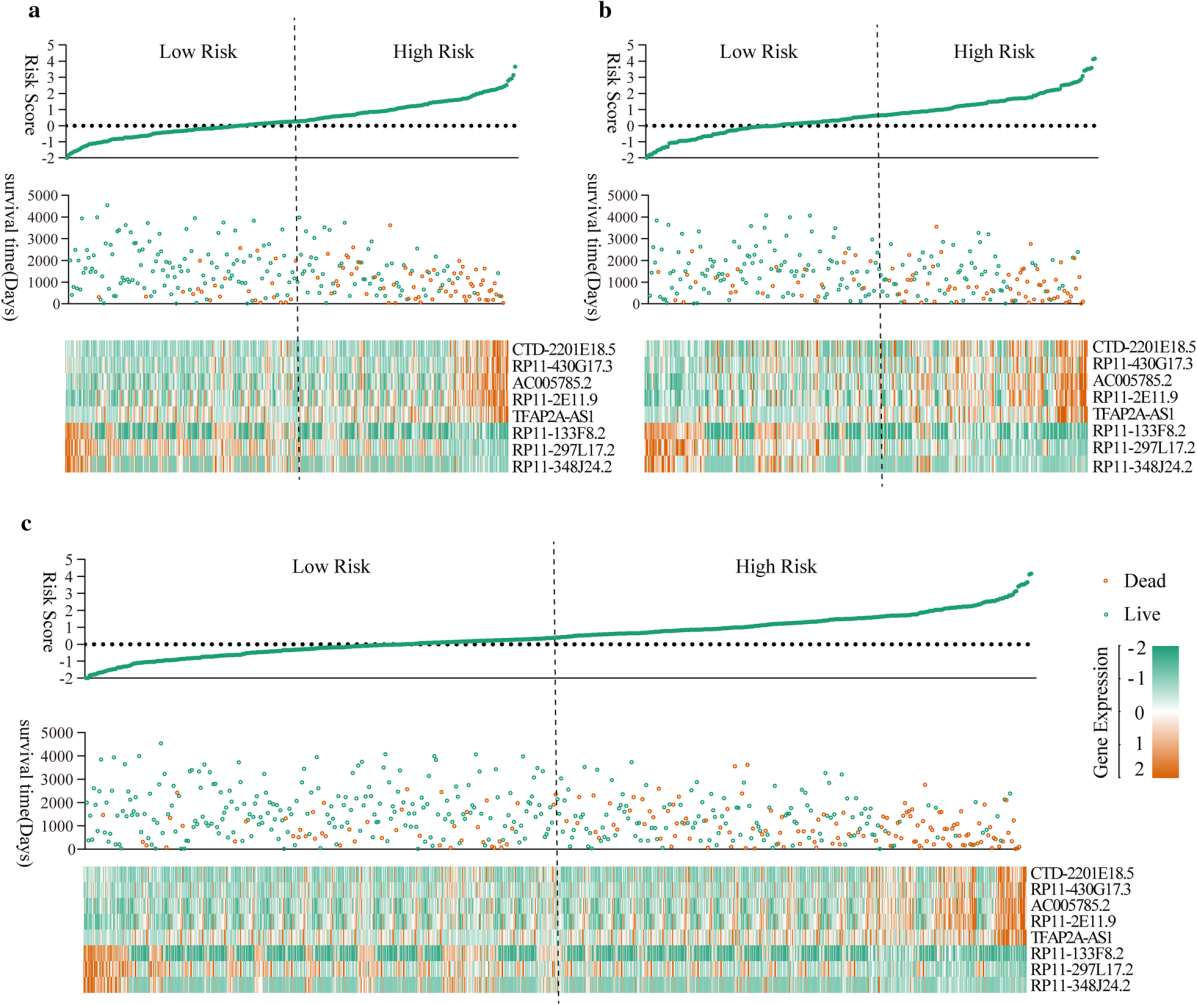
The distribution of the risk score, survival status along with survival times of ccRCC patients and heatmaps of 9 key prognostic lncRNAs. **a** Training group, **b** testing group, **c** training group + testing group

The distribution of the risk score, survival status along with survival time of ccRCC patients and relative expression of the 9 key prognostic lncRNAs were shown in Fig. 2. It indicated that higher risk score predicted shorter survival time of ccRCC patents in all three groups [training group (Fig. 3a), testing group (Fig. 3b) and training group + testing group (Fig. 3c)].

### Survival analysis and time-dependent dynamic ROC

**Fig. 4:**
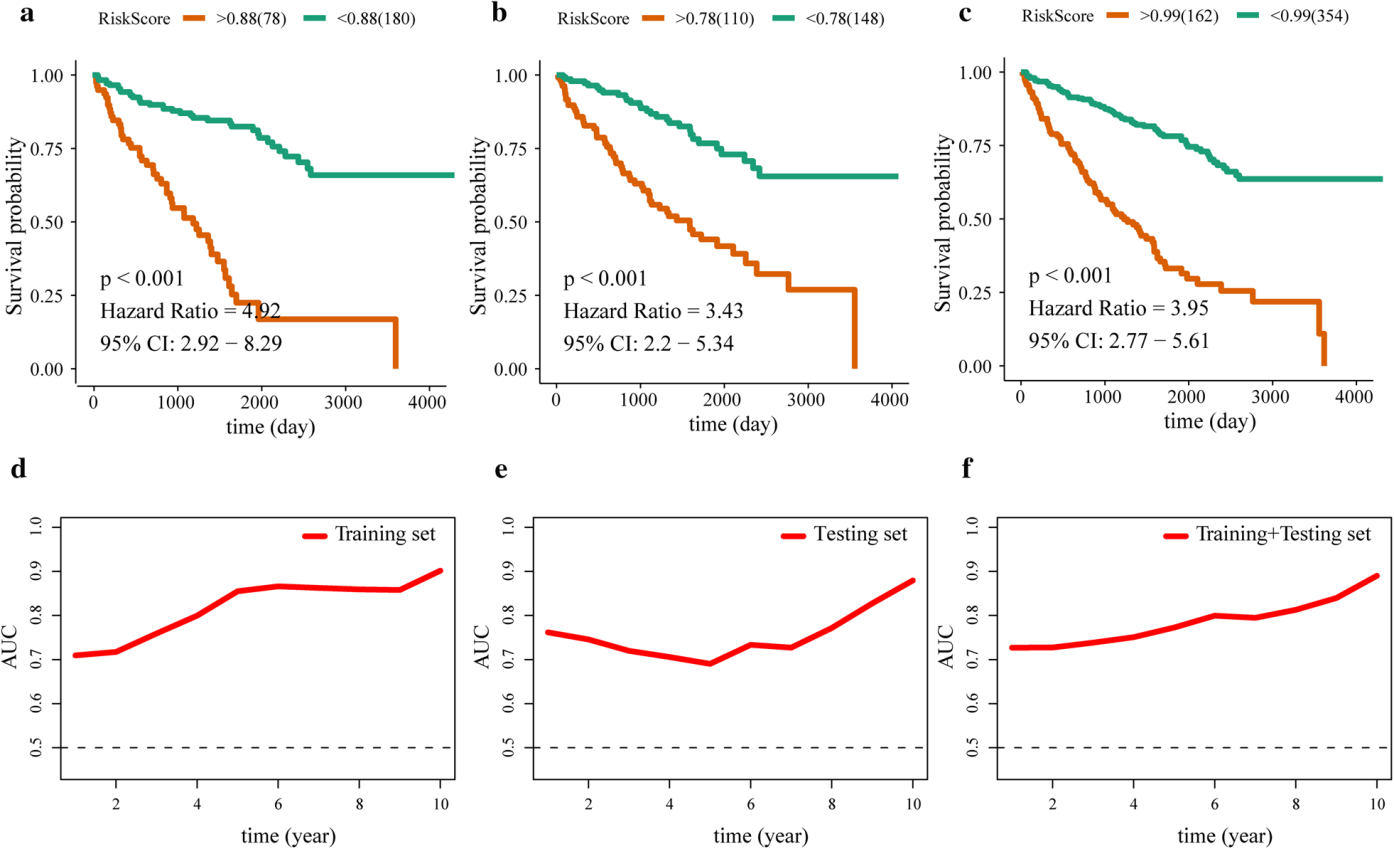
Kaplan–Meier curves and time-dependent dynamic ROCs. Kaplan–Meier curves of training group (**a**), testing group (**b**) and training group + testing group (**c**). time-dependent dynamic ROCs of training group (**d**), testing group (**e**) and training group + testing group (**f**)

OS comparison between patients with high risk score and patients with low risk score was performed by using optimal risk score as cut-off value. The result suggested that patients with higher risk score have shorter OS than patients with low risk score in training group (HR = 4.92, P < 0.001) (Fig. 4a), testing group (HR = 3.43, P < 0.001) (Fig. 4b) and training group + testing group (HR = 3.95, P < 0.001) (Fig. 4c). ROC analysis indicated that the 9-lncRNAs signature had perfect sensitivity and specificity for prognostic prediction of ccRCC patients with AUCs of 1–10 years OS > 0.5 (Fig. 4d–f).

### Clustering analysis of key prognostic lncRNAs

**Fig. 5:**
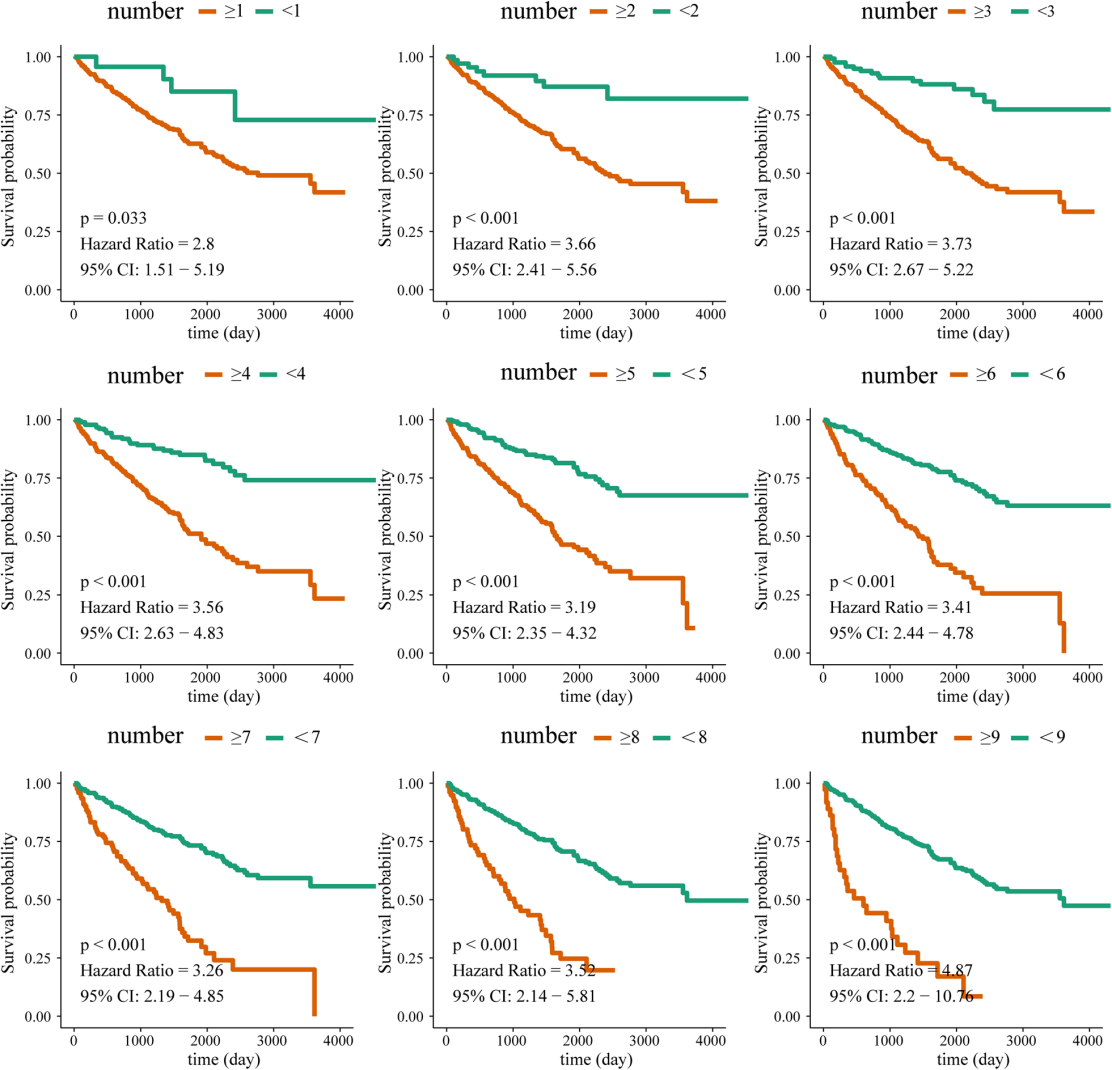
Clustering analysis of key prognostic lncRNAs by using different cut-off values

In order to test the stability of 9-lncRNAs signature, patients of training group + testing group were clustered by using different cut-off values (≥ 1, ≥ 2, ≥ 3, ≥ 4, ≥ 5, ≥ 6, ≥ 7, ≥ 8 and ≥ 9) according to the number of risk factors. As shown in Fig. 5, Kaplan–Meier curves of 9 clusters were all with HR > 1 with significant P values < 0.05, and it suggested that patients with more risk factors would have a poorer prognosis.

### The 9-lncRNAs signature have good prediction ability for both localized ccRCC and advanced ccRCC

**Fig. 6:**
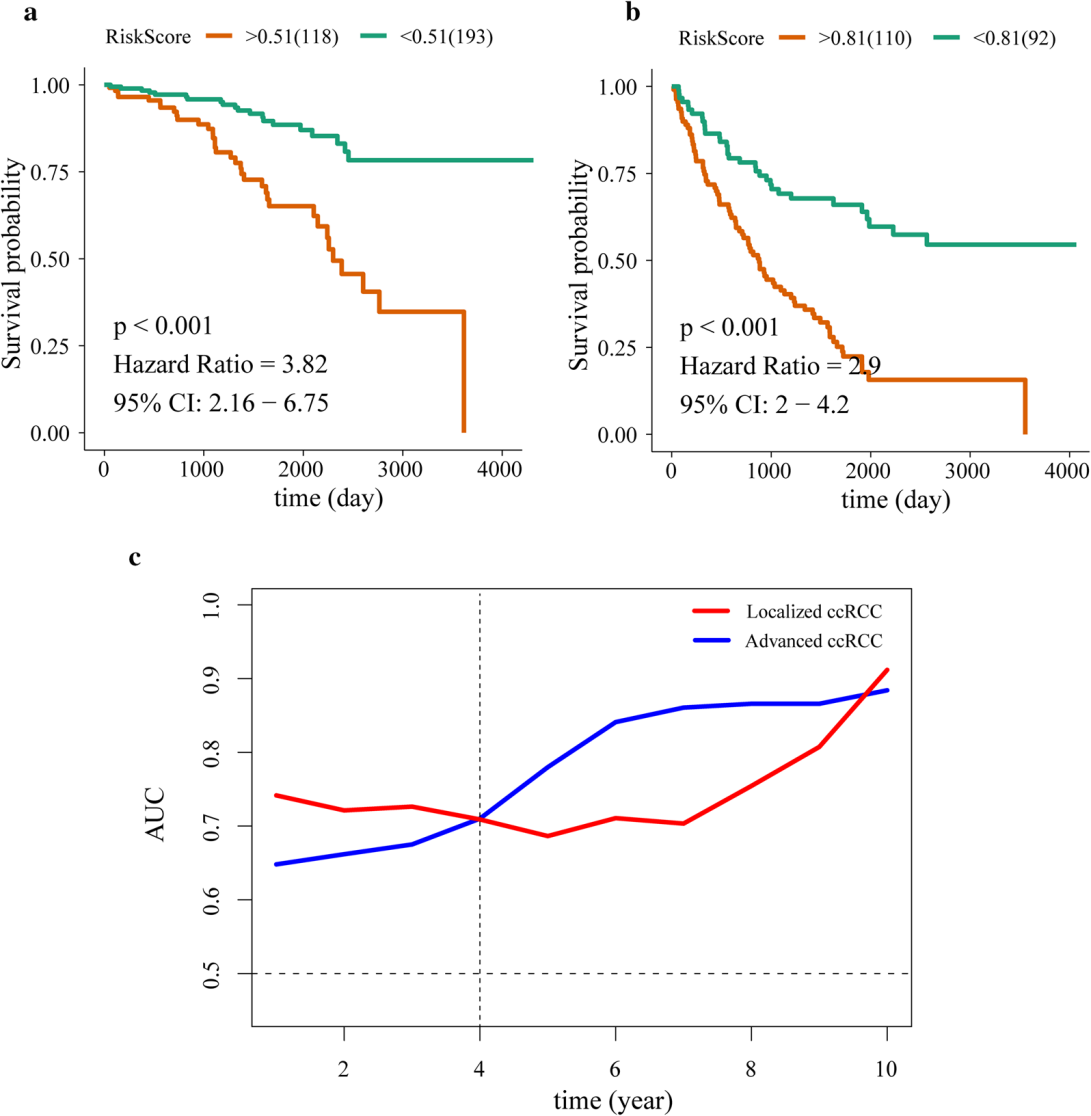
Survival analysis of localized ccRCC patients (**a**) and advanced ccRCC patients (**b**), and time-dependent dynamic ROCs (**c**) for testing sensitivity and specificity of 9-lncRNA signature

Kaplan–Meier curves of localized ccRCC and advanced ccRCC were showed in Fig. 6 by using optimal risk score as cut-off value. Higher risk score was closely associated with poorer prognosis among localized ccRCC patients (Fig. 6a) and advanced ccRCC patients (Fig. 6b), and AUCs of 1–10 years OS in two stages were all above 0.5. Interestingly, the 9-lncRNAs signature had better prediction ability for long term OS (> 4 year) in advanced ccRCC and had better prediction ability for short term OS (< 4 year) in localized ccRCC (Fig. 6c).

### Advanced ccRCC patients with low risk are more sensitive to gemcitabine and sorafenib

**Fig. 7:**
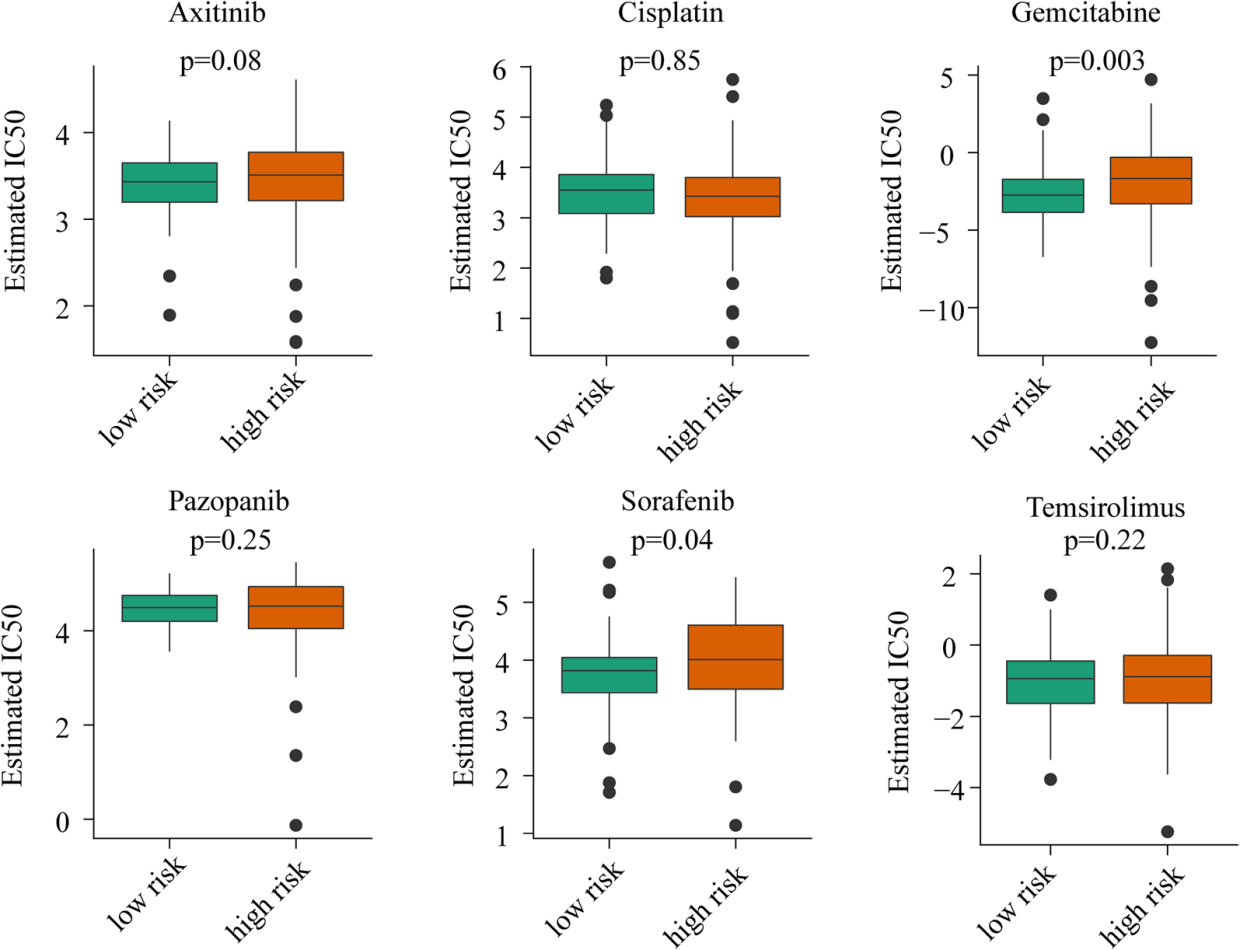
Estimated half-maximal inhibitory concentration (IC50) of high risk group and low risk group for antitumor durgs: axitinib, cisplatin, gemcitabine, pazopanib, sorafenib and temsirolimus

Using median risk score as cut-off value, we observed a significantly different estimated IC50 for gemcitabine and sorafenib between patients with low risk score and patients with high risk score. As shown in Fig. 7, estimated IC50 of patients with low risk were lower than that of patients with high risk for gemcitabine (P = 0.003) and sorafenib (P = 0.04). However, drug sensitivity of advanced ccRCC patients for axitinib, cisplatin, pazopanib, and temsirolimus between high risk and low risk has no significant difference.

### Identification of the 9-lncRNAs signature associated biological pathways

**Fig. 8:**
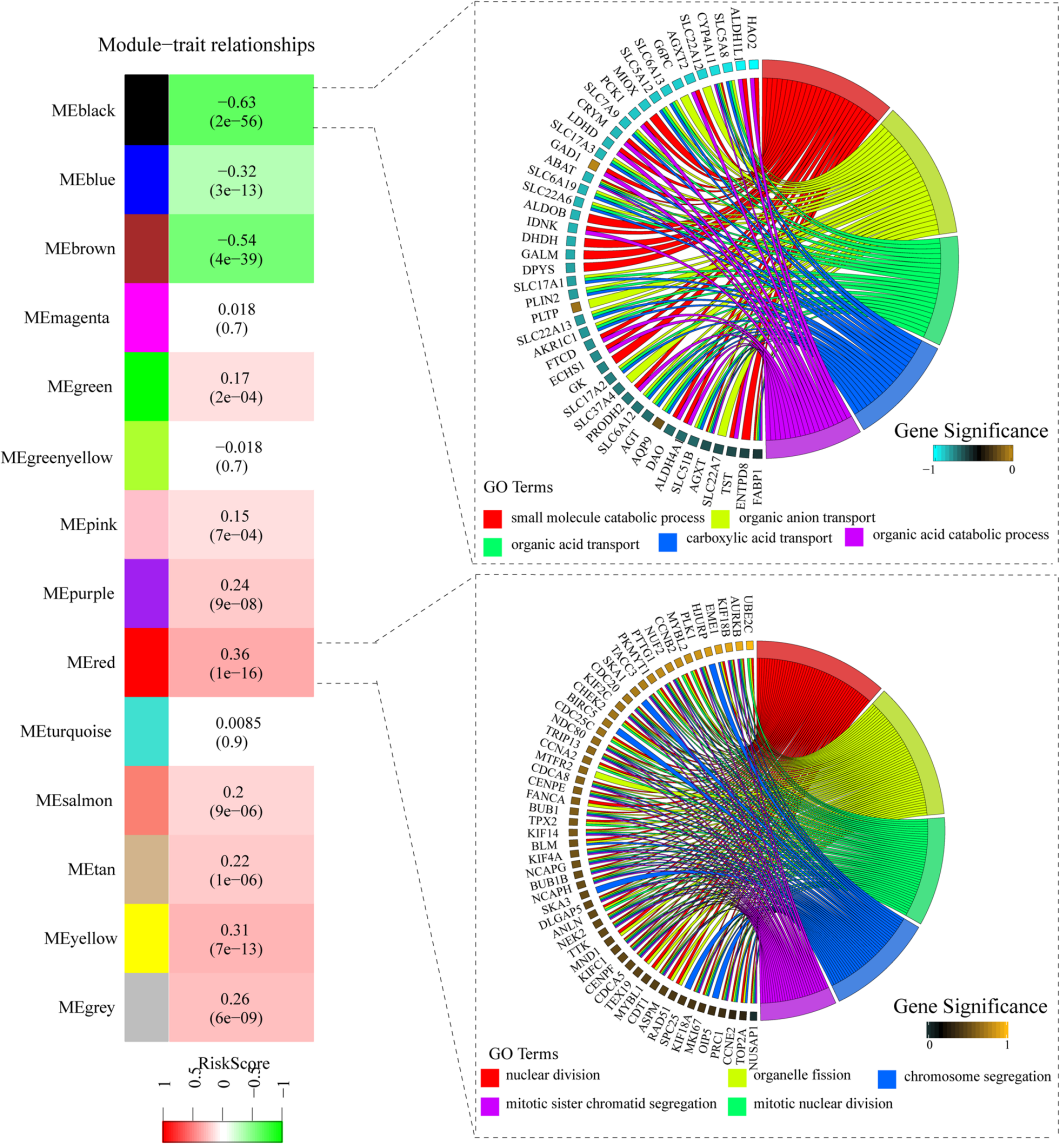
Weight gene co-expression analysis and function enrichment analysis

As depicted above, the 9-lncRNAs signature had a strong discriminatory power for prognosis of ccRCC patients, therefore this signature might be closely associated with biological pathways of ccRCC. 4530 differently expressed mRNAs were used to construct 14 similar expression modules by average linkage clustering. The relevance with P value between each module and 9-lncRNAs signature was listed in every module (Fig. 8). The most negative related module (black module, R = − 0.63, P = 2*E−56) and positive related module (red module, R = 0.36, P = 1*E−16) were selected out for gene enrichment analysis. Genes in black module were mainly enriched in molecular transport associated pathways such as small molecule catabolic process, organic anion transport, organic acid transport, carboxylic acid transport and organic acid catabolic process, and genes in black module were mainly enriched in cell division associated pathways such as nuclear division, organelle fission, mitotic nuclear division, chromosome segregation, mitotic sister chromatid segregation.

### The 9-lncRNA signature is an independent prognostic factor in ccRCC

By χ^2^-test or Fisher’s exact test, we found that the 9-lncRNAs signature was significantly associated with tumor grade, T stage, N stage, M stage, TNM stage and survival status of ccRCC patients (Table 3). Then, relationships between prognosis and clinicopathological characteristics were analyzed by using Cox proportional hazard regression model (Table 4). Univariate analysis showed that 9-lncRNAs signature, age, T stage, N stage, M stage, TNM stage and grade were significantly correlated with OS. While multivariate analysis showed that only 9-lncRNAs signature, age and M stage remained significantly associated with OS. The results indicated that 9-lncRNAs signature was a fine prognostic predictor which was independent of TNM staging system.

**Table 3:** Relationship between clinicopathological characteristics and risk score calculated by using the 9-lncRNAs signature

Factor	Risk score^a^		P value
	Low	High	
Gender			
Male	175 (67.83%)	163 (63.18%)	0.266
Female	83 (32.17%)	95 (36.82%)	
Age			
> 60	120 (46.51%)	137 (53.10%)	0.135
≤ 60	138 (53.49%)	121 (46.90%)	
Grade			
G1 + 2	139 (54.51%)	93 (36.76%)	< 0.001
G3 + 4	116 (45.49%)	160 (63.24%)	
N stage			
N0	111 (98.23%)	123 (89.78%)	0.008
N1	2 (1.77%)	14 (10.22%)	
T stage			
T1 + 2	193 (98.23%)	136 (89.78%)	0.007
T3 + 4	65 (1.77%)	122 (10.22%)	
M stage			
M0	233 (90.66%)	193 (77.51%)	< 0.001
M1	24 (9.34%)	56 (22.49%)	
TNM stage			
I + II	185 (71.98%)	126 (49.22%)	< 0.001
III + IV	72 (28.02%)	130 (50.78%)	
Status			
Live	214 (82.95%)	133 (51.55%)	< 0.001
Dead	44 (17.05%)	125 (48.45%)	

^a^Median expression of risk score was used as cut-off value to cut the patients into high risk group and low risk group

**Table 4:** Cox proportional hazard regression analysis for overall survival of ccRCC patients

Factor	Univariate		Multivariate	
	HR (95% CI)	P value	HR (95% CI)	P value
Age				
> 60 vs ≤ 60	1.462 (1.175–1.82)	0.001	1.369 (1.013–1.851)	0.041
Gender				
Male vs female	0.984 (0.787–1.23)	0.887	–	–
T stage				
T3 + 4 vs T1 + 2	2.282 (1.835–2.839)	< 0.001	1.238 (0.665–2.304)	0.500
N stage				
N1 vs N0	2.287 (1.432–3.651)	0.001	1.045 (0.626–1.746)	0.866
M stage				
M1 vs M0	2.913 (2.337–3.631)	< 0.001	2.171 (1.499–3.143)	< 0.001
TNM stage				
III + IV vs I + II	2.603 (2.072–3.27)	< 0.001	1.148 (0.573–2.299)	0.697
Grade				
G3 + 4 vs G1 + 2	2.028 (1.584–2.596)	< 0.001	1.334 (0.918–1.94)	0.131
Risk score				
≥ Median vs < median	2.595 (2.03–3.318)	< 0.001	2.721 (1.854–3.991)	< 0.001

### Nomogram based on 9-lncRNA signature for prognostic prediction of ccRCC patient

**Fig. 9:**
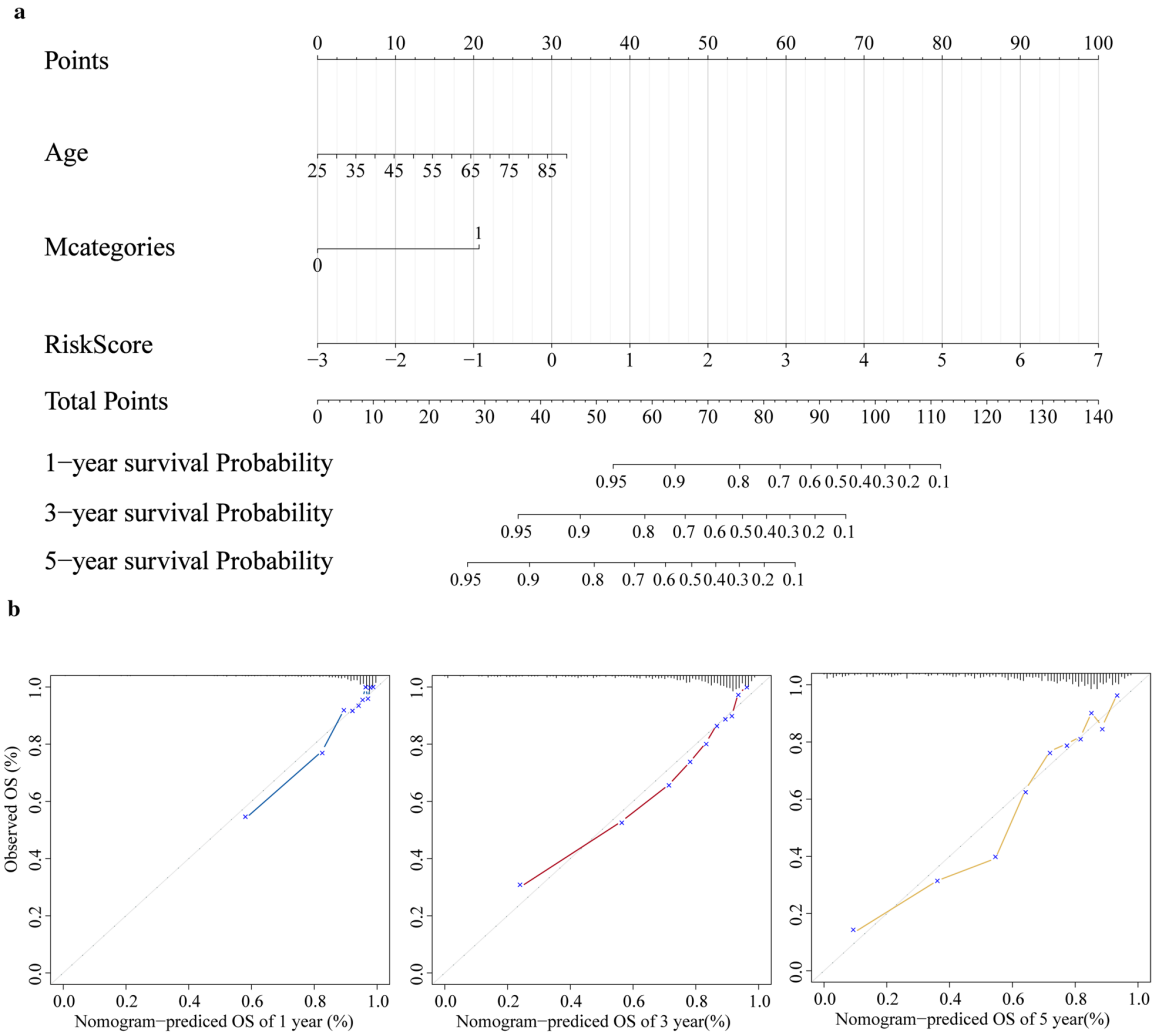
Nomogram and calibration plot. **a** Nomogram based lncRNAs signature, age and M Stage for 1-, 3- and 5-year OS prediction. **b** Calibration plot for agreement test between 1-, 3- and 5-year OS prediction and actual observation

A graphic nomogram including the lncRNAs signature, age and M stage was constructed to predict 1-, 3- and 5-year survival probability of ccRCC patients by using rms R package (Fig. 9a). The C-index of the nomogram was up to 0.79 (95% confidence interval 0.75–0.82), and it meant that the nomogram had a good ability to discriminate patients of poor prognosis from patients of favor prognosis. Meanwhile, the calibration plot showed that 1-, 3- and 5-year survival probability predicted by the nomogram had optimal agreement with actual observation (Fig. 9b).

## Discussion

As we known, ccRCC is a complex tumor caused by intricate genetic and molecular alterations, and some of these alterations are closely associated with the prognosis of ccRCC patients [26]. However, TNM staging system failed to consider these genetic alterations of ccRCC, and it made TNM staging system not perfect for accurate prognostic prediction of ccRCC patients [5]. In this study, we constructed a 9-lncRNAs prognostic signature (RP13-463N16.6, CTD-2201E18.5, RP11-430G17.3, AC005785.2, RP11-2E11.9, TFAP2A-AS1, RP11-133F8.2, RP11-297L17.2 and RP11- 348J24.2) by using robust likelihood-based survival model [19]. Meanwhile, χ^2^-test or Fisher’s exact test found the 9-lncRNAs signature was highly related to tumor grade, T stage, N stage, M stage, TNM stage and survival status of ccRCC patients. And multivariate analysis revealed that the 9-lncRNAs signature, age and M stage were independent prognostic indicators for ccRCC patients. Interestingly, we also found the lncRNA signature could predict therapeutic response of two drugs (gemcitabine and sorafenib), and the signature would provide the reference for guiding clinical use of anti-tumor drugs. Finally, a concise nomogram consisted of the 9-lncRNAs signature, age and M stage was developed for prognostic prediction of ccRCC patients.

In this study, we identified 9 key prognostic lncRNAs of ccRCC patients from TCGA, however, no study had reported about specific biological mechanism of these lncRNAs except TFAP2A-AS1. In a previous study, TFAP2A-AS1 was reported as a tumor suppressor which was associated with better prognosis of breast cancer. The study found that TFAP2A-AS1 acted as miRNA sponge for miR-933 which could degrade smad2 mRNA, and could inhibit proliferation and invasion of breast cancer cell [27]. However, in our study, survival analysis of TFAP2A-AS1 showed that high expression of TFAP2A-AS1 was related to poor prognosis of ccRCC patients. Because of varying lncRNAs’ biofunction and tumor heterogeneity, lncRNA can alter biological behaviors of different tumors toward different directions [28–30]. So, it is not surprising that TFAP2A-AS1 has opposite prognostic effect on breast cancer and ccRCC patients. So far, no study has reported about functions of other 8 lncRNAs, and further study in exploring function of these prognostic lncRNAs in ccRCC are needed in the future.

WGCNA is a data exploratory tool which can identify relationships between external characteristics and high throughput data such as gene microarray, RNA-seq, proteomics data and so on [24]. In order to make clear relationships between the 9-lncRNAs signature and molecular biological mechanism of ccRCC, WGCNA was performed to seek gene modules associated with the 9-lncRNAs signature. Two gene modules, which were most associated with the 9-lncRNAs signature, were identified, and gene enrichment analysis were performed to explore functions of two modules. Interestingly, genes in black module (negatively correlated) mainly enriched in pathways of acid and anion transport and genes in red module (positively correlated) mainly enriched in pathways of cell division and proliferation. As we known, normal tissue of kidney could transport useless metabolite out of our body and maintain water electrolyte balance of our body [31]. So, biological pathways in tumor with low risk score is more like that in normal tissue than that in tumor with high risk score, meanwhile, tumor with high risk score has stronger proliferative ability than tumor with low risk score. In summary, the 9-lncRNAs signature is useful to evaluate differentiated degree of tumor, with low risk score indicating well-differentiated and high risk score indicating poor-differentiated.

lncRNA signatures are novel prognostic systems which made prediction of clinic outcome simpler and more cost-saving, lncRNAs could be fast detected by polymerase chain reaction (PCR) by specific primers. So far, several lncRNAs score systems have been identified for outcome prediction of tumors such as gastric cancer [32–35], lung cancer [36–38], hepatocellular carcinoma [39–41] and so on, and these signatures provide promising biomarkers for prognostic prediction and therapeutic targets for tumor therapy. lncRNAs signatures have also been developed for RCC patients, for example, a 6-lncRNA prognostic signature was developed based on RNA-seq data from TCGA and could precisely predict survival for patients among three RCC subtypes: ccRCC, papillary renal cell carcinoma and chromophobe renal cell carcinoma [42]. Another lncRNA signature named RCClnc4 scores was proved to have higher accuracy for assessing risk of localized ccRCC patients than the TNM staging system and SSIGN score [43]. However, as we known, clinical characteristics such as age, pathological stage, tumor size and tumor grade can also affect prognosis of tumor patients. It would be more accurate for prognostic prediction of RCC patients if these lncRNAs score systems could include these clinical characteristics.

Nomograms are widely used prognostic tools which can generate an individual probability for tumor patients according to corresponding clinical parameters. Nomograms can integrate diverse prognostic variables regardless of continuous variables or discontinuous variables and are user-friendly for clinician judgment [44]. In this study, we got a 9-lncRNAs signature which was highly associated with prognosis of ccRCC patients and independent of TNM staging system. And a prognostic nomogram including 9-lncRNAs signature and clinical characteristics such as age and distant metastases of tumor was developed for prognostic prediction. These clinical characteristics are not influenced by inter-observer variabilities and can be got easily by medical history inquiry, image and pathological examination.

However, our study still has some limitations. First, the nomogram was created based on one cohort obtained from TCGA, and it would be better if validated by other cohorts. Second, some potential clinical characteristics such as blood biochemistry and nutritional status were ignored. Third, LncRNAs are dynamic in body and this characteristic may made the signature not stable, multiple site biopsies would be helpful to improve stability of lncRNA signature. Despite these limitations, this is the first nomogram which was based on lncRNAs signature and provided a new methodology of developing prognostic score system for ccRCC patients. This nomogram can easily separate patients with poor prognosis from patients with good prognosis by performing PCR. And clinicians can develop more individualized treatment regimens for patients with different prognosis, this will make treatment more targeted and save more public health resources. Meanwhile, this nomogram consists of objective indicators which prevent prognostic prediction from inter-observer variabilities of different pathologists.

## Conclusions

In summary, a 9-lncRNAs signature associated with prognosis of ccRCC patients were constructed, and a promising prognostic nomogram was developed based on a 9-lncRNAs signature for 1-, 3- and 5-year OS prediction of ccRCC patients in this study. This nomogram did not depend on pathological stage and variables easily effected by inter-observer variabilities. It will help clinicians make treatment decision more easily and accurately in the future.

## Data Availability

The datasets supporting the conclusions of this article are included within the article.

## Abbreviations

RCC: renal cell carcinoma
ccRCC: clear cell renal cell carcinoma
UICC: International Union Against Cancer
AJCC: American Joint Committee on Cancer
lncRNAs: long noncoding RNAs
UCA1: urothelial carcinoma associated 1
MALAT1: metastasis-associated lung adenocarcinoma transcript 1
MRCCAT1: metastatic renal cell carcinoma-associated transcript 1
RPKM: reads per kilobase per million mapped reads
TPM: transcripts per kilobase of exon model per million mapped reads
DEGs: differentially expressed genes
FC: fold change
OS: overall survival
HR: hazard rate
AICs: Akaike’s information criterions
ROC: receiver operating characteristic curve
AUC: ROC curve area under the curve
WGCNA: weighted gene co-expression network analysis
C-index: Harrell’s concordance index
